# P-379. The Effect of SARS-CoV-2 Vaccination on HIV Viral Load in Patients Under Bictegravir/Tenofovir Alafenamide/Emtricitabine Therapy: A Retrospective Observational Study

**DOI:** 10.1093/ofid/ofaf695.597

**Published:** 2026-01-11

**Authors:** Giuseppe Pipitone, Giacomo Ciusa, Stefano Agrenzano, Francesco Di Lorenzo, Claudia Imburgia, Caterina Sagnelli, Antonio Cascio, Chiara Iaria

**Affiliations:** ARNAS Civico, Palermo, Sicilia, Italy; ARNAS Civico, Palermo, Sicilia, Italy; ARNAS Civico, Palermo, Sicilia, Italy; ARNAS Civico, Palermo, Sicilia, Italy; ARNAS Civico, Palermo, Sicilia, Italy; Vanvitelli University, Naples, Campania, Italy; Policlinic Hospital of Palermo, Palermo, Sicilia, Italy; ARNAS Civico, Palermo, Sicilia, Italy

## Abstract

**Background:**

The aim of our study is to evaluate the impact of SARS-CoV-2 vaccination on HIV viremia in patients treated under bictegravir-based therapy.

Although cases of transiently detectable HIV viremia after the Severe Acute Respira- tory Syndrome COronaVirus-2 (SARS-CoV-2) vaccine in a patient on antiretroviral therapy (ART) have been described by other authors, the association between vaccination and viremic blips is not unambiguously defined.

**Figure 1.**
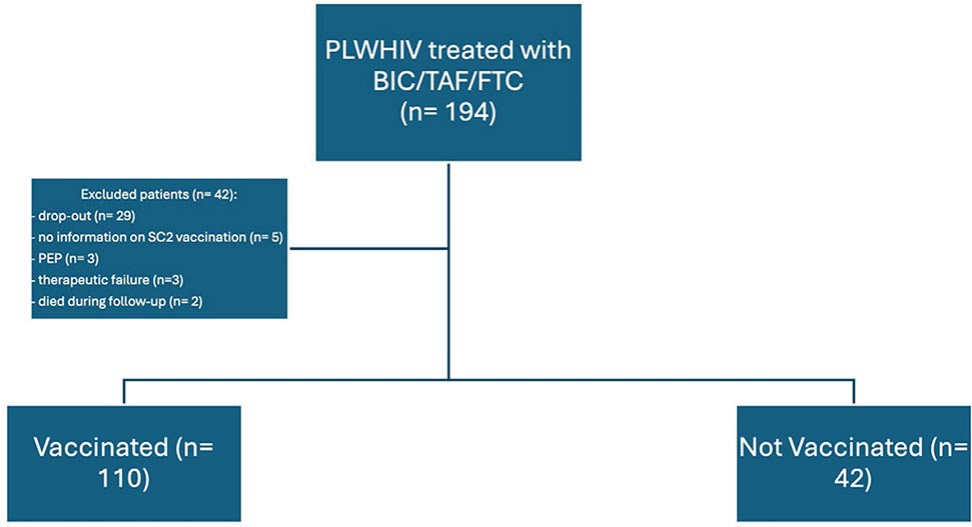
Patient’s enrollement flow chart. Patients’ enrollment. PLWHIV: people living with HIV. HIV: Human immunodeficiency virus. BIC: bictegravir. TAF: Tenofovir alafenamide. FTC: Emtricitabine. SC2: SARS-CoV-2. PEP: post-exposure vaccination

**Figure 2.**
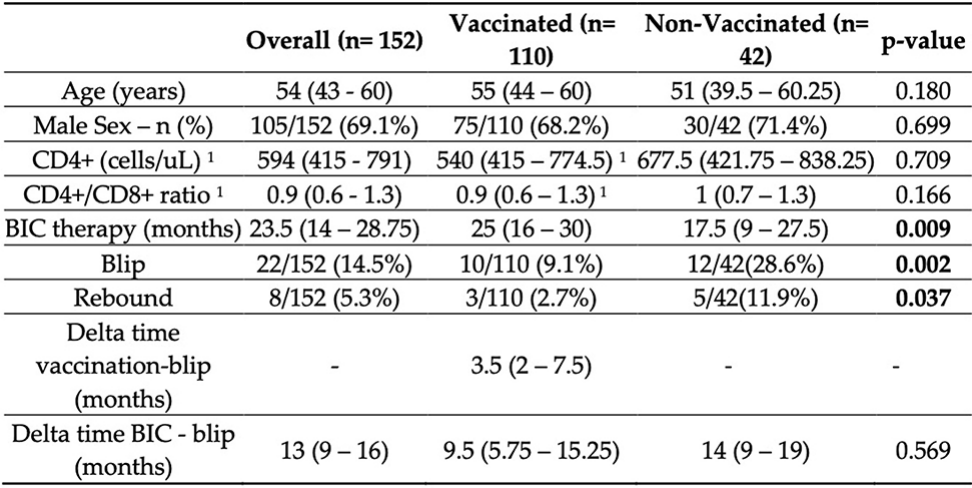
Patients’ characteristics are described Age, sex, lymphocyte CD4+ values, CD4+/CD8+ 106 ratio, the time difference between vaccination and blip, the time difference from the start of therapy 107 with Bictegravir and the blip were collected. BIC= Bictegravir/Tenofovir alafenamide/Emtricitabine. 108 Data are presented as median and IQR 25-75%, or number and percentage (%). Delta time 109 vaccination-blip: time difference between vaccination and blip observation. Delta time BIC-blip: 110 time difference between starting BIC therapy and blip observation. 1 data missing for one patient

**Methods:**

We conducted a retrospective observational study in a tertiary hospital, analyzing data from 194 patients treated with BIC/TAF/FTC between 2020 and 2022. Among 194 patients, 152 was included (Fig.1). Patients were divided into two groups: “vaccinated” (110/152) and “unvaccinated” (42/152) against SARS-CoV-2. The outcomes considered were the presence of “blips” (detectable viremia ≥ 20 copies/mL), “rebound” (viremia ≥ 50 copies/mL), and virological failures.

**Results:**

The overall population was predominantly composed of males (105/152, 69.1%), without statistically significant differences between the two groups (p = 0.699), and the median age was 54 years (IQR 43–60, 95% CI) without differences between the two groups (p = 0.180). Fig. 2

Patients who presented a blip were 22/152 (14.5%), with a lower rate of blips in the vaccinated group compared to the non-vaccinated group (respectively, 9.1% vs. 28.6%, p = 0.002). We observed rebound overall in 8/152 (5.3%), with a lower rate in the vaccinated group compared to non-vaccinated (2.7% vs. 11.9%, p = 0.037). Among the 22 patients who had presented with a blip, the blip “event” occurred with a median of 3.5 months (IQR 2–7.5, 95% CI). To evaluate the risk of a blip we performed a logistic regression analysis for confounding factors, showing a reduced risk of blips in the vaccinated group (OR 3.8, 95% CI 1.4–9.8)

**Conclusion:**

Our data suggest that SARS-CoV-2 vaccination may stimulate an immune response that enhances CD4+ and CD8+ cell function, contributing to a reduction in the number of blips and maintaining good viro-immunological control in patients with HIV, supporting the importance of vaccination in this population.

**Disclosures:**

All Authors: No reported disclosures

